# Interdisciplinary collaborative teaching in vascular surgery training: A randomized trial of radiologist-surgeon partnership in China

**DOI:** 10.1371/journal.pone.0341266

**Published:** 2026-01-27

**Authors:** Xin Li, Wei Hu

**Affiliations:** 1 Department of Radiology, Sichuan Provincial People’s Hospital, University of Electronic Science and Technology of China, Chengdu, China; 2 Department of Vascular Surgery, Sichuan Provincial People’s Hospital, University of Electronic Science and Technology of China, Chengdu, China; Makere University College of Health Sciences, UGANDA

## Abstract

This randomized controlled trial evaluates an innovative interdisciplinary teaching model co-led by radiologists and vascular surgeons within China’s standardized residency training program. Forty trainees were randomized into two groups: one receiving collaborative teaching, which included joint lectures, radiologist-attended ward rounds, and interdisciplinary case conferences; and the other undergoing traditional vascular single-discipline training. The experimental group exhibited superior performance in CT interpretation accuracy (92.0% vs. 71.0%, P < 0.01), diagnostic accuracy (87.0% vs. 67.0%, P < 0.01), treatment plan rationality (mean 4.40 ± 0.75 vs. 3.65 ± 0.88, P < 0.01), and communication skills (median 43.00 vs. 33.00, P < 0.0001). These findings validate that structured interdisciplinary collaboration effectively bridges the gap between radiology and clinical practice, suggesting a paradigm shift in vascular surgical education.

## Introduction

In modern medical education systems, standardized residency training serves as the cornerstone for cultivating clinical physicians, with continuous innovation in teaching models remaining a critical focus in medical education reform [1]. The rapid advancement of medical imaging technologies, particularly the widespread adoption of multi-slice spiral CT angiography (CTA) and digital subtraction angiography (DSA), has profoundly transformed the diagnostic and treatment paradigms for vascular surgical diseases [2]. According to the National Vascular Surgery Technology Development Report 2024, over 85% of vascular surgeries now require preoperative imaging assessments for precise planning, placing higher demands on residents’ imaging interpretation skills and collaborative competence across disciplines [3]. However, traditional single-discipline teaching models exhibit significant limitations in fostering integrated clinical reasoning, particularly in scenarios requiring real-time imaging-clinical correlation, which may hinder residents’ ability to formulate comprehensive treatment plans in complex vascular pathologies [4,5].

In response to these challenges, the International Institute for Medical Education (IIME) explicitly advocates for “organ-system-based, problem-oriented collaborative teaching” in the 21st Century Medical Education Declaration [6]. China’s Residency Training Standards 2023 also identifies “multidisciplinary collaborative competence” as a core competency for vascular surgery residents [3]. Against this backdrop, a collaborative teaching model between radiologists and vascular surgeons has emerged. By breaking down disciplinary barriers and integrating imaging expertise with clinical practice, this model provides an innovative pathway for cultivating interprofessional medical professionals [7].

This collaborative model is grounded in established learning theories, particularly social constructivism and experiential learning theory. Social constructivism posits that knowledge is co-constructed through social interaction and collaboration with more knowledgeable others [8]. The dual-mentor setup, bringing together radiologists and surgeons, creates a “community of practice” where trainees actively build understanding through dialogue and shared problem-solving. Furthermore, the model embodies Kolb’s experiential learning cycle, where residents concrete experience in ward rounds and case discussions is followed by reflective observation, abstract conceptualization during joint lectures, and active experimentation in formulating treatment plans [9]. This theory-driven approach ensures that learning is not passive but an active, contextualized, and reflective process, thereby bridging the theory-practice gap more effectively than traditional methods.

Current research on collaborative teaching primarily focuses on the fields of oncology, stomatology, and nursing [10–12]. leaving vascular surgery understudied. This study innovatively constructs a “dual-mentor” collaborative teaching system through a trinity framework of joint lectures, case-based discussions, and hands-on practice. The aim is to address the disconnect between imaging education and clinical practice in traditional training. Using a randomized controlled trial (RCT) approach, we comprehensively evaluate residents’ capabilities across four critical dimensions — imaging interpretation, disease diagnosis, treatment strategy formulation, and doctor – patient communication — in order to furnish evidence – based support for promoting medical education reform.

## Materials and methods

### Study design

(1) Design: Prospective Randomized Controlled Trial (RCT), using a two-arm, parallel-group design.
(2) Scenario setting: The study was conducted at Sichuan Provincial People’s Hospital from 18/07/2024–31/03/2025.
(3) Participants: A total of 40 vascular surgery residents were recruited for the study, with a mean age of 26.3 years and 80% male. Participants were randomly assigned to either the experimental group or the control group, with 20 people in each group.
(4) Sample Size Calculation: Sample size was estimated based on a pilot study, assuming a 20% improvement in diagnostic accuracy with 80% power and α = 0.05, yielding a minimum of 18 participants per group. We recruited 20 per group to account for potential attrition.
(5) Ethical approval: The study was approved by the Institutional Review Board of Sichuan Provincial People’s Hospital (IRB No.2024−429). All participants provided written informed consent prior to enrollment. The consent process included a detailed explanation of the study purpose, procedures, potential risks, and benefits. Participants were assured of voluntary participation and the right to withdraw at any time without consequences.

### Interventions

#### Experimental group.

(1) Joint Lectures: A series of lectures were held, with 50% of the content delivered by radiologists and 50% by vascular surgeons. These lectures focused on integrating imaging findings with clinical management.
(2) Weekly Collaborative Case Conferences: Every week, residents participated in case conferences where radiologists and vascular surgeons jointly discussed complex cases, highlighting the interdisciplinary approach to patient care.
(3) Radiologist-Involved Ward Rounds: Radiologists accompanied vascular surgery teams during ward rounds, providing real-time imaging interpretation and facilitating bedside teaching. The radiologists involved were all senior attending physicians with over 5 years of experience in vascular imaging and prior involvement in medical education.

#### Control group.

Conventional vascular surgery teaching was provided, which included standard lectures, case discussions, and ward rounds led solely by vascular surgeons. The entire teaching content was independently completed by vascular surgeons.

### Outcome measures

#### Assessment of CT image interpretation accuracy.

The accuracy of residents’ CT image interpretations was evaluated using 5 randomly selected CT cases, each representing distinct vascular pathologies (e.g., aortic aneurysms, aortic dissections, arteriosclerotic occlusions). These cases were derived from anonymized patient data and validated by senior radiologists to ensure clinical relevance and imaging complexity. Residents in the experimental and control groups independently reviewed the cases under standardized conditions, with a 5-minute time limit per case.

Structured scoring criteria:

0 points: Incorrect diagnostic conclusion.
1 point: Accurate diagnostic conclusion.

Total scores for each resident ranged from 0–5 points, with group totals scaled proportionally to a 100-point system. This rigorous, objective evaluation framework ensured effective assessment of residents’ ability to analyze imaging findings.

#### Assessment of diagnostic accuracy.

**Case Selection:** Diagnostic accuracy was assessed using 25 standardized cases(including 5 acute/chronic limb ischemia, 5 aortic aneurysms, 5 aortic dissections, 5 visceral artery diseases, and 5 venous disorders) that included clinical histories, physical examination findings, and imaging data. Residents were required to integrate all available information to establish a final diagnosis.

**Standard Development:** standard diagnoses were independently established by senior vascular surgeons and radiologists through consensus.

**Assessment Protocol:** Participants: 40 residents (20 experimental group, 20 control group) each evaluated 5 unique cases (100 assessments per group).

**Evaluation Metrics**: Per-case accuracy: Binary score (correct/incorrect) compared to the gold standard. Per-resident accuracy: Proportion of correct diagnoses (0–5).

#### Assessment of treatment plan rationality.

The reasonability of treatment plans is assessed using a validated 5-point Likert scale specifically designed for vascular surgery scenarios. The scale ranges from 1 point, indicating “completely unreasonable”, to 5 points, indicating “completely reasonable”. The assessment criteria were developed through a Delphi consensus process involving extensive participation from senior vascular surgeons and radiologists, to ensure consistency with current clinical guidelines and interdisciplinary practice standards.

**Assessment Method:** All participants were randomly assigned a case for evaluation. Based on the provided case information, each participant formulated a treatment plan. The rationality of each plan was evaluated by a consensus panel comprising one senior vascular surgeon and one senior radiologist. The panel discussed each plan against the predefined criteria to assign a single score on the 5-point Likert scale.

**Assessment Criteria:** 5 points: The treatment plan fully adheres to evidence-based guidelines, takes into full consideration the patient’s individual factors (such as comorbidities, imaging findings, etc.), and reflects interdisciplinary considerations (such as preoperative imaging correlation analysis, comparison between minimally invasive and open surgical options, etc.).

4 points: The treatment plan is generally reasonable but has minor room for adjustment, such as suboptimal imaging follow-up plans or slight deviations from guideline recommendations.

3 points: The treatment plan is marginally acceptable but has significant limitations, such as incomplete risk-benefit analysis or errors in imaging interpretation that affect decision-making.

2 points: The treatment plan is clearly unreasonable and has major defects, such as performing contraindicated procedures or ignoring critical imaging findings.

1 point: The treatment plan is completely unreasonable or harmful, such as containing life-threatening errors or ethical violations.

#### Communication skills assessment.

**Assessment Tool:** The Communication Skills Inventory (CSI) was used for assessment. This tool, adapted from Amber W. Trickey [13], comprises 10 core items and was validated in previous medical education studies for comprehensively measuring interpersonal skills in clinical settings. These items cover key areas such as Clarity & Professionalism, Empathy & Listening Skills, Feedback Skills & Non-verbal Communication, Completeness of Information & Adaptability, and Confidence & Respect.

**Scoring Method:** Each item was rated on a 5-point Likert scale, ranging from 1 to 5, where 1 indicated “Strongly Disagree” and 5 indicated “Strongly Agree”. Higher scores indicated stronger communication skills in the corresponding area.

**Assessment Process and Assessors:** The assessment was conducted by trained senior clinicians and nursing staff to ensure objectivity. Assessors were blinded to the group allocation of participants to eliminate bias. They observed residents during simulated patient encounters and real-life clinical rounds.

**Scoring Calculation:** The total score for each participant was the sum of the scores for the 10 items, ranging from 10 to 50. A maximum score of 50 indicated excellent communication skills in all assessed areas.

This tool allowed us to comprehensively assess the communication skills of residents while keeping the assessment process concise and efficient.

### Statistical analysis

The data were analyzed using GraphPad Prism. Continuous variables (e.g., Likert-scale scores for treatment plan rationality) were compared using independent t-tests, while Likert-scale scores for communication skills were analyzed using Mann-Whitney U tests. Categorical variables (e.g., accuracy of CT interpretation and diagnostic accuracy) were evaluated using Chi-square tests. A P-value less than 0.05 was considered statistically significant.

## Results

The following are the main results of a prospective randomized controlled trial (RCT) designed to evaluate the effectiveness of a collaborative teaching model involving radiologists and vascular surgeons in the standardized training of vascular surgery residents:

1. Accuracy of CT Image Interpretation: Table 1 presents the performance of the two groups of residents in terms of the accuracy of CT image interpretation.

**Table 1:** Accuracy of ct image interpretation.

Group	Number of Cases	Number of Correct Interpretations	Accuracy (%)	P-value
Experimental	100	92	92.0	<0.01
Control	100	71	71.0	

The accuracy of CT image interpretation among residents in the experimental group was significantly higher than that of the control group, with an accuracy rate of 92.0% compared to 71.0% in the control group (P < 0.01). This result indicates that the collaborative teaching model effectively improves residents’ ability in image interpretation.

1. Diagnostic Accuracy: Table 2 compares the diagnostic accuracy between the two groups of residents.

**Table 2:** Comparison of diagnostic accuracy.

Group	No. of Residents	Total Assessments	Correct Diagnoses	Accuracy (%)	Odds Ratio (95% CI)	P-value
Experimental	20	100	87	87.0	3.30(1.74–6.25)	<0.01
Control	20	100	67	67.0	Reference	—

The experimental group achieved significantly higher diagnostic accuracy (87.0% vs. 67.0%, Odds Ratio = 3.3, P < 0.01). Mixed-effects regression confirmed the collaborative model significantly improved diagnostic odds after controlling for case complexity.

1. Rationality of Treatment Plans: Table 3 presents the scores for the rationality of treatment plans between the two groups of residents.

**Table 3:** Rationality of treatment plans.

Group	n	Mean Score (±SD)	P-value
Experimental	20	4.40 ± 0.75	<0.01
Control	20	3.65 ± 0.88	

The mean score for the rationality of treatment plans among residents in the experimental group was 4.40 ± 0.75, significantly higher than the 3.65 ± 0.88 in the control group (P < 0.01). This result suggests that the collaborative teaching model helps residents formulate more rational treatment plans.

1. Communication Skills: Table 4 presents the scores for communication skills between the two groups of residents.

**Table 4:** Communication Skills (CSI Scale).

Group	n	Median	P-value
Experimental	20	43.00	<0.0001
Control	20	33.00	

The median score for communication skills among residents in the experimental group was 43.00, significantly higher than the 33.00 in the control group (P < 0.0001, Maximum score: 50). This data indicates that the collaborative teaching model effectively improves residents’ communication skills, which are crucial for improving patient-doctor relationships and team collaboration.

## Discussion

The results of this prospective randomized controlled trial (RCT) underscore the significant advantages of a collaborative teaching model that integrates radiologists and vascular surgeons in the standardized residency training of vascular surgery residents. This innovative approach not only bridges the gap between imaging expertise and clinical practice but also enhances residents’ overall competence in critical domains such as CT image interpretation, diagnostic accuracy, treatment plan rationality, and communication skills. In the following sections, we delve deeper into the implications of these findings, compare them with existing literature, discuss the potential mechanisms underlying the observed improvements, and explore the limitations and future directions of this study.

### Enhanced imaging interpretation and diagnostic accuracy

The collaborative teaching model demonstrated a remarkable 21.0% increase in CT interpretation accuracy compared to the traditional single-discipline approach. This finding aligns with previous studies that have highlighted the benefits of interdisciplinary collaboration in improving diagnostic accuracy. For instance, Peloneet al. [14] demonstrated that interprofessional practice-based interventions significantly enhance clinical outcomes through shared expertise. Similarly, Bhattacharya et al. [15] emphasized that competency-based medical education frameworks, which prioritize interdisciplinary integration, are essential for bridging knowledge-practice gaps. The enhanced imaging interpretation skills observed in our study can be attributed to the joint lectures and weekly collaborative case conferences, which provided residents with a comprehensive understanding of how imaging findings correlate with clinical management [16]. By exposing residents to diverse perspectives and encouraging them to think critically about imaging data, the collaborative model fosters a deeper understanding of the complex interplay between imaging features and clinical presentations.

Moreover, the diagnostic accuracy of residents in the experimental group was significantly higher (87.0% vs. 67.0% in the control group). This improvement suggests that the collaborative teaching model not only enhances imaging interpretation skills but also translates these skills into better clinical decision-making. The involvement of radiologists in ward rounds and case discussions likely contributed to this outcome by providing real-time feedback and facilitating the application of imaging knowledge in clinical contexts. This real-world application of imaging expertise is crucial for developing a well-rounded vascular surgery resident who can confidently interpret images and make accurate diagnoses.

### Rationality of treatment plans

The collaborative teaching model also led to a significant increase in the rationality of treatment plans proposed by residents. The mean score for treatment plan rationality in the experimental group was 4.40 ± 0.75, compared to 3.65 ± 0.88 in the control group. This finding indicates that residents exposed to the collaborative model were better able to formulate rational and evidence-based treatment plans. The interdisciplinary nature of the collaborative teaching model likely played a pivotal role in this improvement. By working closely with radiologists and vascular surgeons, residents gained a more holistic understanding of patient care, enabling them to consider a broader range of diagnostic and treatment options.

This improvement resonates with the principles outlined in the Residency Training Standards (2023 Edition) [3], which advocate for multidisciplinary competency development. The Delphi consensus process used in our assessment criteria aligns with methodologies recommended by Hong et al. [17] for ensuring rigor in interdisciplinary research. Furthermore, the integration of interdisciplinary tumor boards have been shown to optimize treatment planning [18].

The rationality of treatment plans is a critical aspect of clinical competence, as it reflects a resident’s ability to integrate knowledge from multiple disciplines and apply it to individual patient cases. The collaborative model’s emphasis on joint lectures, case discussions, and hands-on practice likely contributed to this enhancement by providing residents with opportunities to practice formulating and refining treatment plans in a supportive and interdisciplinary environment.

### Communication skills

Communication skills are essential for effective patient care and team collaboration. The collaborative teaching model significantly improved residents’ communication skills, with a mean score of 43.40 ± 1.23 in the experimental group compared to 32.95 ± 1.15 in the control group.

This 10.45% improvement is particularly noteworthy, as effective communication is crucial for establishing trust with patients, conveying complex medical information, and coordinating care among healthcare professionals, consistent with findings from Sargeant [19], who highlighted that interprofessional education fosters empathy and adaptability in communication. The use of structured assessment tools like the Communication Skills Inventory (CSI) aligns with validated frameworks for evaluating clinical communication [20].

The improvement in communication skills can be attributed to the interdisciplinary nature of the collaborative model, which encourages residents to interact with and learn from radiologists and vascular surgeons. Radiologists, in particular, bring a unique perspective to patient care, as they are often responsible for explaining imaging findings to patients and other healthcare providers. By working closely with radiologists, residents likely gained insights into effective communication strategies, such as using clear and concise language, employing visual aids, and demonstrating empathy and professionalism.

The significant improvements observed across all assessed domains can be understood through the lens of the social constructivist and experiential learning theories that underpin our collaborative model. The “dual-mentorship” structure is a direct application of Vygotsky’s concept of the “More Knowledgeable Other,” where residents learn not only the technical skills of their own specialty but also the diagnostic reasoning and imaging interpretation schema of their radiology colleagues. This social mediation of knowledge accelerates the development of integrated clinical reasoning. Similarly, the model fully engages Kolb’s experiential learning cycle. The joint case conferences and radiologist-involved ward rounds provide rich, concrete clinical experiences. The real-time feedback from radiologists and surgeons facilitates reflective observation and abstract conceptualization, helping residents form mental models that connect imaging findings to clinical decisions. Finally, residents apply these refined mental models through active experimentation in diagnosing cases and devising treatment plans. This theory-based, iterative process of experience, reflection, and application likely explains the profound enhancement in both technical competence and communication skills.

### Potential mechanisms and implications

Several mechanisms could explain residents’ competence improvement after the collaborative teaching intervention. Firstly, the interdisciplinary nature of the model, with dual – mentorship, exposes residents to diverse perspectives. This aligns with the “organ - system - based, problem - oriented” approach [6]. Collaborating with mentors from different specialties broadens residents’ understanding of patient care. Secondly, joint activities like lectures, case discussions, and hands – on practice offer practical learning opportunities. This case – based learning approach resonates with Fleischmann et al.’s [16] emphasis on contextualizing imaging knowledge in clinical scenarios. It enhances residents’ diagnostic and therapeutic skills. Thirdly, real – time feedback and bedside teaching from radiologists during ward rounds likely accelerate clinical skill acquisition, as supported by previous research [21]. Residents can observe and emulate experienced professionals’ communication and decision – making skills.

The implications of these findings are significant for medical education and patient care. By adopting a collaborative teaching model, residency programs can better prepare vascular surgery residents for the complex and multidisciplinary nature of modern vascular care. Improved imaging interpretation and diagnostic accuracy, rational treatment planning, and effective communication skills are all critical for providing high-quality patient care and achieving optimal patient outcomes.

### Limitations and future directions

Despite its strengths, this study has several limitations. First, the single-center design limits the generalizability of the findings to other institutions, a challenge also noted in similar RCTs by Zwarenstein et al. [22]. Future studies should explore the scalability of the collaborative teaching model in different settings and with larger sample sizes, adopting multi-center designs [23]. Second, the short follow-up period prevents us from assessing the long-term impact of the intervention on residents’ competence and patient outcomes. Longitudinal follow-up, as proposed in competency-based education frameworks [24], is needed to examine the sustained effects of the collaborative model over time. Following the study, all control group participants were offered the same collaborative teaching modules to ensure equitable access to the educational innovation.

Future research should also explore the potential benefits of extending the collaborative teaching model to other specialties and stages of medical education. For example, incorporating radiologists into the training of medical students or residents in related fields, such as interventional radiology or cardiology, could further enhance interdisciplinary collaboration and improve patient care. This expansion aligns with the Residency Training Standards (2023 Edition) [3], which emphasize multidisciplinary competency development across medical disciplines.

**In conclusion**, the collaborative teaching model involving radiologists and vascular surgeons effectively integrates imaging expertise with clinical practice, improving resident competence in critical domains. This approach should be adopted in residency programs to meet the demands of modern vascular care and enhance the overall quality of patient care. By fostering interdisciplinary collaboration and providing residents with a comprehensive understanding of patient care, the collaborative model has the potential to transform medical education and improve patient outcomes.

## Supporting information

S1 Table Raw data of Table 1. (XLSX)

S2 Table Raw data of Table 2. (XLSX)

S3 Table Raw data of Table 3. (XLSX)

S4 Table Raw data of Table 4. (XLSX)
